# Psychotic-like experiences in community-dwelling young people in hong kong: Preliminary finding from the hong kong youth epidemiological study of mental health (HKYES)

**DOI:** 10.1192/j.eurpsy.2021.560

**Published:** 2021-08-13

**Authors:** C.S. Wong, C.C. Chan, S.K. Chan, C.L. Hui, E.H. Lee

**Affiliations:** Department Of Psychiatry, The University of Hong Kong, HK, Hong Kong PRC

**Keywords:** community, youth people, psychotic-like experiences, mental health

## Abstract

**Introduction:**

Psychotic-like experiences (PLEs) are often referred to as psychotic experiences, such as hallucinations and delusions, in the absence of a psychotic disorder. PLEs as part of the continuum of psychosis suggested that healthy population can endorse PLEs without having significant distress or impairment which would warrant them a clinical diagnosis. While PLEs are usually associated with psychotic disorders, previous research has also shown the link between PLEs and many other mood symptoms.

**Objectives:**

The present study aims to identify PLEs in community youths and explore the underlying risk and protective factors.

**Methods:**

This is an ongoing study in which young people aged 15-24 were recruited from community through a random stratified sampling method. Sociodemographic, lifestyle, functioning, and other psychosocial factors were assessed in a face-to-face structured interview. In particular, PLEs were assessed using the Composite International Diagnostic Interview Screening Scales (CIDI-SC). Six domains of lifetime PLEs were measured, including auditory and visual hallucination, thought insertion/ withdrawal, delusion of control and reference, and persecutory delusions.

**Results:**

To date, 746 participants were recruited and of these, 3.2% of them has endorsed lifetime PLEs. Results showed that significantly higher depressive, anxiety and stress scores were found in those who has PLEs (p<0.001), and additionally, these scores significantly predicted the presence of PLEs in regression models (p<0.001).

**Conclusions:**

Our preliminary findings highlighted the inter-related phenomena between PLEs and mood symptoms. Further investigation is needed to examine the likelihood of PLEs in predicting psychosis over time.

